# Generation of permanent neonatal diabetes mellitus dogs with glucokinase point mutations through base editing

**DOI:** 10.1038/s41421-021-00304-y

**Published:** 2021-10-12

**Authors:** Xiaomin Wang, Yanhui Liang, Jianping Zhao, Yuan Li, Shixue Gou, Min Zheng, Juanjuan Zhou, Quanjun Zhang, Jidong Mi, Liangxue Lai

**Affiliations:** 1 CAS Key Laboratory of Regenerative Biology, Guangdong Provincial Key Laboratory of Stem Cell and Regenerative Medicine, South China Institute for Stem Cell Biology and Regenerative Medicine, Guangzhou Institutes of Biomedicine and Health, Chinese Academy of Sciences, Guangzhou, 510530 China; 2 Beijing SINOGENE Biotechnology Co., Ltd, Beijing, 102200 China; 3 University of Chinese Academy of Sciences, Beijing, 100049 China; 4 Bioland Laboratory (Guangzhou Regenerative Medicine and Health Guangdong Laboratory), Guangzhou, 510005 China; 5 Research Unit of Generation of Large Animal Disease Models, Chinese Academy of Medical Sciences (2019RU015), Guangzhou, 510530 China; 6 Present Address: School of Life Sciences, Westlake University, Shilongshan Road No. 18, Cloud Town, Xihu District, Hangzhou Zhejiang, 310024 China

**Keywords:** Gene expression profiling, Mechanisms of disease

Dear Editor,

Permanent neonatal diabetes mellitus (PNDM) in humans can be caused by the homozygous nullification of glucokinase (GCK), which is a key rate-limiting enzyme in glucose metabolism in pancreatic β cells and hepatocytes and is considered as the “glucose sensor” for the regulation of insulin secretion^1–5^. Various mouse models have been generated through global, isoform-specific, or tissue-specific *GCK* gene knockout to help understand the role of GCK in glucose homeostasis. Mice with global homozygous *GCK* knockout or pancreatic β-cell specific *GCK* knockout presented severe hyperglycemia and died within a few days of birth^6–9^, even treated with insulin or glibenclamide^7^. However, patients with GCK-PNDM could live to adulthood when treated with insulin^10^. Suitable PNDM models with homozygous *GCK* mutations for mimicking the symptoms of human patients with GCK-PNDM authentically are currently unavailable. Dogs, which are omnivorous animals like humans, are considered as a highly valuable large animal model for human metabolic diseases, such as diabetes^11^. Point mutation is the most frequent mutation pattern associated with the deficient expression of a gene. Previously, gene-edited dogs with random indels at the targeted site had been generated by using the CRISPR/Cas9 system^12,13^, which was unsuitable for generating models of genetic disease caused by single-nucleotide mutations. However, approaches for generating point mutations in dogs have not been reported. Here, for the first time, by utilizing newly developing base-editing technology named BE3 system, we attempted to generate a dog model of PNDM that contained homozygous *GCK* point mutations.

We first validated the base editing efficiency of the BE3 system in canine embryonic fibroblasts (CEFs) with 5 different genes (*GCK*, *MSTN*, *IL2RG*, *RAG1*, and *RAG2*) and 4 different sites (*GCK-1*, *GCK-2*, *GCK-3*, and *GCK-4*) of the same gene. BE3 system was able to mediate C-to-T conversion within different genes and sites of CEFs efficiently varying from 22.2%–56.3% (Supplementary Fig. S1a). Both monoallelic and biallelic mutants of C-to-T conversion were found in all the targeted sites except for *GCK-1* and *GCK-3* (Supplementary Fig. S1a–c). The base editing efficiency of a combination of 2 genes (*RAG1* and *RAG2*), 3 genes (*RAG1*, *RAG2*, and *IL2RG*), and 2 sites within the same gene (*GCK*-2 and *GCK*-3) was 37.5%, 15.0%, and 45.5% respectively (Supplementary Fig. S2a–c).

A total of 56 zygotes were collected and injected with a mixture of BE3 mRNA and *GCK*-4 sgRNA, which was located in exon 2 of the *GCK* gene (Fig. 1a). Seventeen puppies were obtained, and four (190619, 190627, 190628, and 190761) of them exhibited C-to-T conversion within *GCK*-4 target site (Supplementary Fig. S3a). PCR amplification and sequencing results showed that the 4 positive dogs were homozygotes with C-to-T mutation at the target site (Supplementary Fig. S3b, c). Tissues of heart, liver, lung, kidney, pancreas, brain, and muscle were collected from 2 (190619 and 190761) dead and 1 (190627) sacrificed PNDM dogs for further genotyping tests. The results also showed that the biallelic mutant with C-to-T conversion at the target site existed in the all of the tissues of the 3 dogs (Supplementary Fig. S3d, e). Deep sequencing results showed 3 positive dogs (190619, 190627, and 190761) were homozygotes with more than 99.9% of the sequencing reads edited at the target site in the livers and pancreases (Fig. 1b and Supplementary Fig. S4a, b). While the other positive dog (190628) was a chimera with 90.5% of sequencing reads modified in the genome from ear punch tissue (Fig. 1b and Supplementary Fig. S4b). For detection of sgRNA-dependent off-target, we selected 15 potential off-target sites (OTS1-15), which were 2 or 3 nucleotide mismatches at the target site (Supplementary Table S1) of the 4 GCK-PNDM dogs. We performed next-generation sequencing (NGS) to further detect the off-target in these OTSs. The results showed that 2 potential OTSs (OTS2 and OTS7) were found with C > T/G > A mutations in the 4 (50%, 2/4 and 75%, 3/4, respectively) base-edited dogs (Supplementary Fig. S4c, d), but no other off-target mutations were detectably induced at OTSs in *GCK* mutant dogs compared with the 3 WT samples. In addition to the sgRNA-dependent DNA off-target, sgRNA-independent DNA off-target as well as RNA off-target of BE3 has also been reported^14–16^. Next, we sought to detect sgRNA-independent off-target by using whole-genome sequencing (WGS) analysis. The results showed that no sgRNA-independent off-target was found in the PNDM dogs (Supplementary Fig. 5a–d). For detection of RNA off-target of the PNDM dog, we analyzed the data from RNA-seq of the PNDM (190627) and WT dogs. The results showed that there was no significant difference in the number of de novo SNVs, indels, and proportion of C > U/G > A at the RNA level between the livers from 190627 and WT dogs (Supplementary Fig. 5e–g). It meant that no RNA off-target was found in dogs at 27 weeks.

**Fig. 1:**
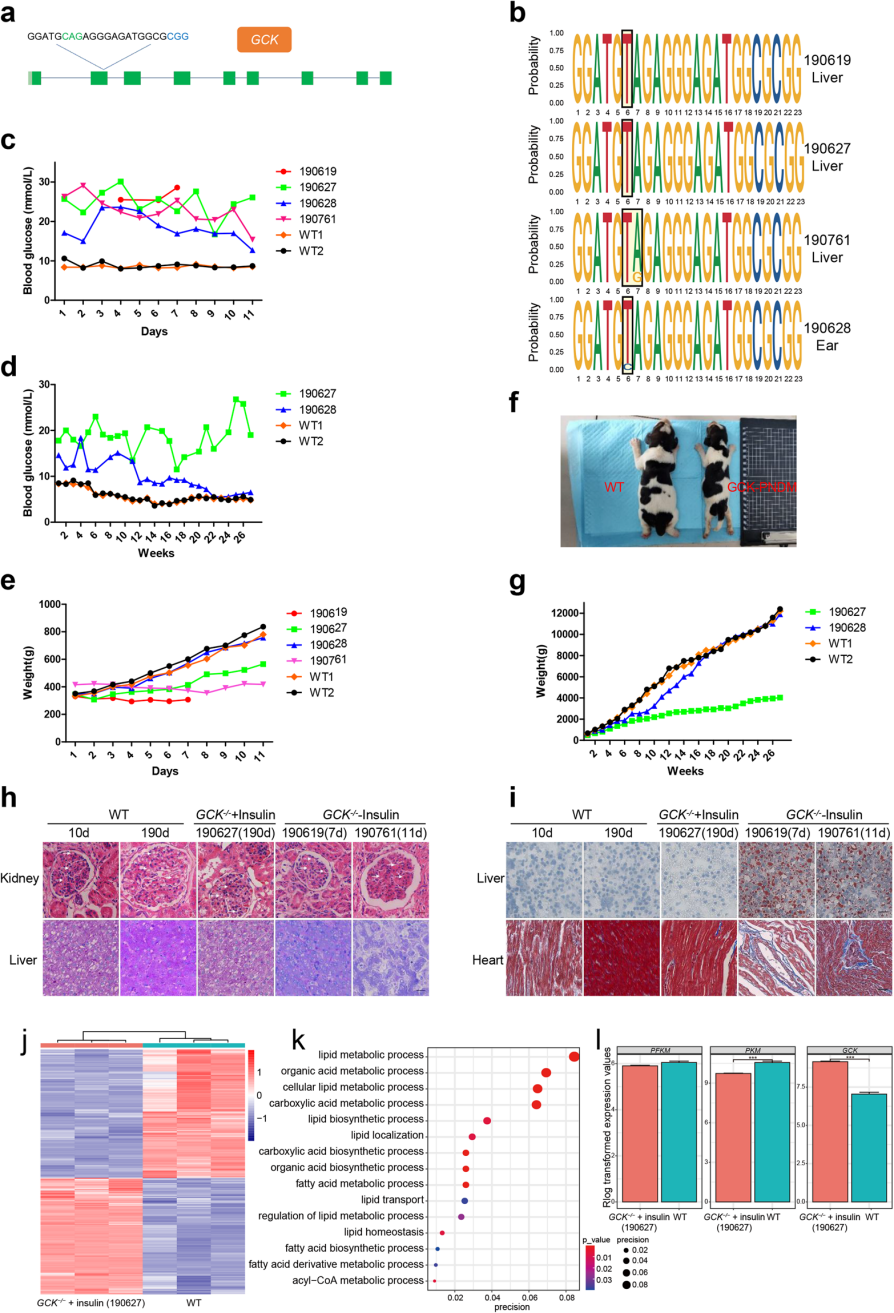
Generation of GCK-PNDM dogs with base editing. **a** The target sequences at the *GCK* locus. Target sequences (black), protospacer adjacent motif (PAM) region (blue), target sites (green). **b** Sequence motif of livers from the GCK-PNDM dogs (190619, 190627, and 190761) and the ear punch tissue from the chimeric dog (190628). **c**, **d** Blood glucose levels of WT (*n* = 2) and the GCK-PNDM dogs (*n* = 4, 190619, 190627, 190628, and 190761) from the 1st day to the 11th day after birth (**c**) and from the 1st week to the 27th week (**d**). **e**–**g** Body weights of WT (*n* = 2) and the GCK-PNDM dogs (*n* = 4, 190619, 190627, 190628, and 190761) from the 1st day of birth to the 11th day after birth (**e**) and from the 1st week to the 27th week (**g**). Photographs of WT dog (left) and the insulin untreated GCK-PNDM dog (right: 190619) at the 5th day after birth (**f**). **h**, **i** Histological analysis of WT (10 d and 190 d), *GCK*^*-/-*^ + insulin (190 d), and *GCK*^*-*^^*/-*^ – insulin (7 and 11 d) dogs. H&E staining of kidney sections (top) showed the amount of mesangial matrix and the thickness of the glomerular basement membrane (white arrow). PAS staining of liver sections for analysis of glycogen (purple color) synthesis (**h**). Oil red *O* staining of liver sections (top) for analysis of lipid droplets (red color) accumulation. Masson’s trichrome staining (blue color) of heart sections for analysis of myocardial fibrosis in myocardium (**i**). Scale bar: 80 μm. **j** Hierarchical clustering analysis of liver cells from WT and insulin treated GCK-PNDM dogs. Red and blue represent higher and lower gene expression levels, respectively. Data represented three biological replicates. **k** Gene ontology enrichment analysis of the genes differentially expressed in biological processes between the insulin-treated GCK-PNDM and WT dogs. *P* < 0.05. **l** Bar plots of representative genes involved in the glycolytic pathway. ***P < 0.001; ns, not significant.

The blood glucose levels of the 3 positive dogs (190619, 190761, and 190627) were above 20 mmol/L, which were almost 2 times higher than that of the wild type (WT) dogs at an early age (Fig. 1c). Two of the dogs (190627 and 190628) injected with insulin daily after birth were able to survive for a long time (Supplementary Fig. S6a). While the other 2 cared dogs (190619 and 190761) died at 7 and 11 days after birth without insulin treatment, respectively (Supplementary Fig. S6a). The blood glucose level in the treated homozygous dog (190627) also constantly maintained high with some extend of fluctuations until the dog was sacrificed at 27 weeks after birth (Fig. 1d). The blood glucose level of the treated chimeric dog (190628) was lower than that of the homozygous dogs but still exceeded that of the WT dogs before 11 weeks after birth, decreased gradually, and eventually decreased to normal levels at 23 weeks after birth (Fig. 1d). The birth weights of all the base-edited dogs did not differ from those of the WT dogs. Two untreated homozygous dogs (190619 and 190761) suffered from daily weight loss until death (Fig. 1e, f). The treated homozygous dog (190627) experienced weight gain at a rate that was slower than the weight gain rate of WT dogs (Fig. 1e, g). Similar to that of the WT dogs, the weight of the treated chimeric dog (190628) increased normally from the 1st to 27th week (Fig. 1g). These results demonstrated that the GCK-PNDM dogs had growth retardation.

Hematoxylin and eosin staining results showed that the amount of mesangial matrix and the thickness of the glomerular basement membrane increased in the kidneys of untreated GCK-PNDM dogs (190619 and 190761), and no difference was observed between the treated GCK-PNDM dog (190627) and 2 WT dogs (Fig. 1h). No abnormal histological changes were found in the livers and hearts (Supplementary Figs. S6b–c) of all the 3 GCK-PNDM dogs. Periodic acid-Schiff (PAS) staining results showed that the glycogen in the liver and kidney of the untreated GCK-PNDM dogs (190619 and 190761) had decreased compared with those of the WT dogs. Moreover, the treated dog (190627) had a normal glycogen level (Fig. 1h and Supplementary Fig. S6d). Liver sections stained with Oil red *O* of GCK-PNDM dogs (190619 and 190761) showed the presence of numerous lipid droplets within livers, whereas only a few lipid droplets were found in the livers of the insulin-treated GCK-PNDM dog (190627) and WT dogs (Fig. 1i). Fibrosis was observed in the myocardium of treated and untreated GCK-PNDM dogs by Masson’s trichrome staining, and the abnormal phenotype of the untreated dogs was more severe than that of the treated one (Fig. 1i).

Bulk RNA-seq analysis was performed to analyze the status of glucose and lipid metabolism in the liver of a GCK-PNDM dog (190627). Differential expression analysis revealed that 2362 and 2606 genes were upregulated and downregulated, respectively (Supplementary Fig. S7a) in the GCK-PNDM dog relative to that in the WT dog. The hierarchical clustering of the upregulated and downregulated genes was shown (Fig. 1j). Gene Ontology analysis revealed that the differentially expressed genes were enriched in biological processes such as lipid metabolism, lipid transport, fatty acid metabolism, and acyl-CoA metabolism (Fig. 1k). Genes related to a fatty acid or lipid metabolism were upregulated and genes related to fatty acids or lipids synthesis were downregulated (Supplementary Fig. S7b, c). Genes (*PFKM* and *PKM*) involved in the glycolytic pathway downregulated, but, the expression of the *GCK* was upregulated in the GCK-PNDM dog (Fig. 1l). However, genes involved in the glycogen metabolic process (Supplementary Fig. S7d) and TCA cycle (Supplementary Fig. S7e) in the GCK-PNDM dog were similar to those in the WT dogs. These results demonstrated that glucose metabolism could be maintained at a normal level in the GCK-PNDM dog after long-term insulin injection.

In summary, we first used the BE3 system to generate GCK-PNDM dogs with homozygous point mutations, which exhibited similar features to those of the GCK-PNDM patients. These dogs will provide an ideal animal model for the study of biological mechanisms and the development of novel therapeutic methods for GCK-PNDM.

## Supplementary information

Supplementary information
