# Author Correction: Generation of permanent neonatal diabetes mellitus dogs with glucokinase point mutations through base editing

**DOI:** 10.1038/s41421-021-00348-0

**Published:** 2021-12-09

**Authors:** Xiaomin Wang, Yanhui Liang, Jianping Zhao, Yuan Li, Shixue Gou, Min Zheng, Juanjuan Zhou, Quanjun Zhang, Jidong Mi, Liangxue Lai

**Affiliations:** 1 CAS Key Laboratory of Regenerative Biology, Guangdong Provincial Key Laboratory of Stem Cell and Regenerative Medicine, South China Institute for Stem Cell Biology and Regenerative Medicine, Guangzhou Institutes of Biomedicine and Health, Chinese Academy of Sciences, Guangzhou, 510530 China; 2 Beijing SINOGENE Biotechnology Co., Ltd, Beijing, 102200 China; 3 University of Chinese Academy of Sciences, Beijing, 100049 China; 4 Bioland Laboratory (Guangzhou Regenerative Medicine and Health Guangdong Laboratory), Guangzhou, 510005 China; 5 Research Unit of Generation of Large Animal Disease Models, Chinese Academy of Medical Sciences (2019RU015), Guangzhou, 510530 China; 6 Present Address: School of Life Sciences, Westlake University, Shilongshan Road No. 18, Cloud Town, Xihu District, Hangzhou, Zhejiang, 310024 China

**Keywords:** Gene expression profiling, Mechanisms of disease

Correction to: *Cell Discovery* (2021) 7:92

10.1038/s41421-021-00304-y Published online 12 October 2021

In the original publication of this Correspondence^1^, we made an error in the authors affiliations. We apologize for any inconvenience that it may have caused.

Now we provided a corrected version here about the affiliations of all authors here.

Xiaomin Wang^1,3,4,5,6^, Yanhui Liang^1,3,4,5^, Jianping Zhao^2^, Yuan Li^2^, Shixue Gou^1,3,4,5^, Min Zheng^2^, Juanjuan Zhou^1,4,5^, Quanjun Zhang^1,4,5^, Jidong Mi^2^ and Liangxue Lai^1,4,5^

^1^CAS Key Laboratory of Regenerative Biology, Guangdong Provincial Key Laboratory of Stem Cell and Regenerative Medicine, South China Institute for Stem Cell Biology and Regenerative Medicine, Guangzhou Institutes of Biomedicine and Health, Chinese Academy of Sciences, Guangzhou, 510530, China

^2^Beijing SINOGENE Biotechnology Co., Ltd, Beijing, 102200, China

^3^University of Chinese Academy of Sciences, Beijing, 100049, China

^4^Bioland Laboratory (Guangzhou Regenerative Medicine and Health Guangdong Laboratory), Guangzhou, 510005, China

^5^Research Unit of Generation of Large Animal Disease Models, Chinese Academy of Medical Sciences (2019RU015), Guangzhou, 510530, China

^6^Current address: School of Life Sciences, Westlake University, Shilongshan Road No. 18, Cloud Town, Xihu District, Hangzhou, Zhejiang 310024, China

These authors contributed equally: Xiaomin Wang, Yanhui Liang, Jianping Zhao.

Correspondence: Jidong Mi (jidong-m@sinogene.com.cn) or Liangxue Lai (lai_liangxue@gibh.ac.cn)
