# Supplementary material for: Generation of permanent neonatal diabetes mellitus dogs with glucokinase point mutations through base editing
Source: Cell Discov. 2021 Oct 12;7:92. doi: 10.1038/s41421-021-00304-y (PMC8505425; doi:10.1038/s41421-021-00304-y)
Supplement: Supplementary file 1 — Supplementary information [file 41421_2021_304_MOESM1_ESM.pdf]

a

Target genes	Target sequences	Mutant alleles(Mutated clones/tested clones)		
		Monoallelic mutant(%)	Biallelic mutant(%)	Total mutant(%)
GCK-1	AGCCCAGACAGCCTTGAGTCTGG	8/31(25.8%)	0/31(0.0%)	8/31(25.8%)
GCK-2	CATCCTCCTCAATTGGACCAAGG	6/17(35.3%)	1/17(5.9%)	7/17(41.1%)
GCK-3	AAGCACAAGAAGCTGCCCTTGGG	4/18(22.2%)	0/18(0.0%)	4/18(22.2%)
GCK-4	GGATGCAGAGGGAGATGGCGCGG	10/27(37.0%)	5/27(18.5%)	15/27(55.6%)
MSTN	CGACCAGTACGACGTCCAGAGGG	3/16(18.8%)	6/16(27.5%)	9/16(56.3%)
IL2RG	TGTCCAGCTCCGGGACCCACGGG	1/17(5.9%)	4/17(23.5%)	5/17(29.4%)
RAG1	GGAGCAATCTCCAGCAGTCCCGG	3/10(30.0%)	2/10(20.0%)	5/10(50.0%)
RAG2	TATAGTCAAGGGAGAAGCATGGG	2/16(12.5%)	6/16(37.5%)	8/16(50.0%)

b

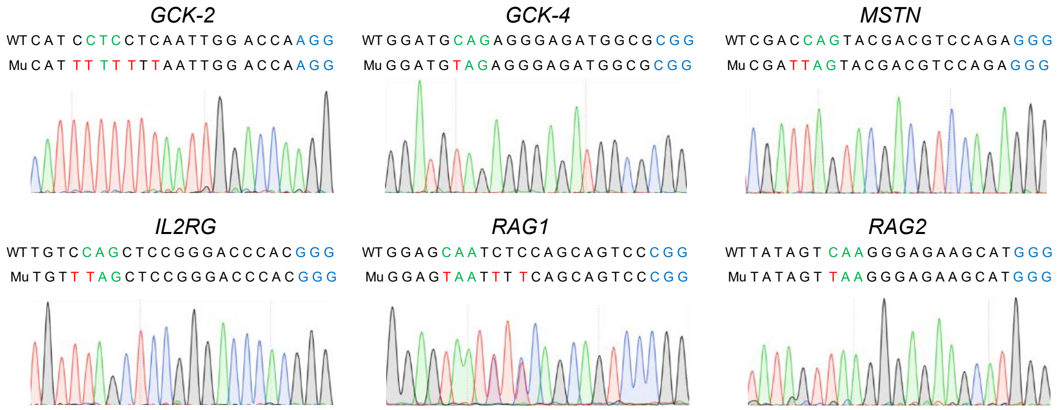

c

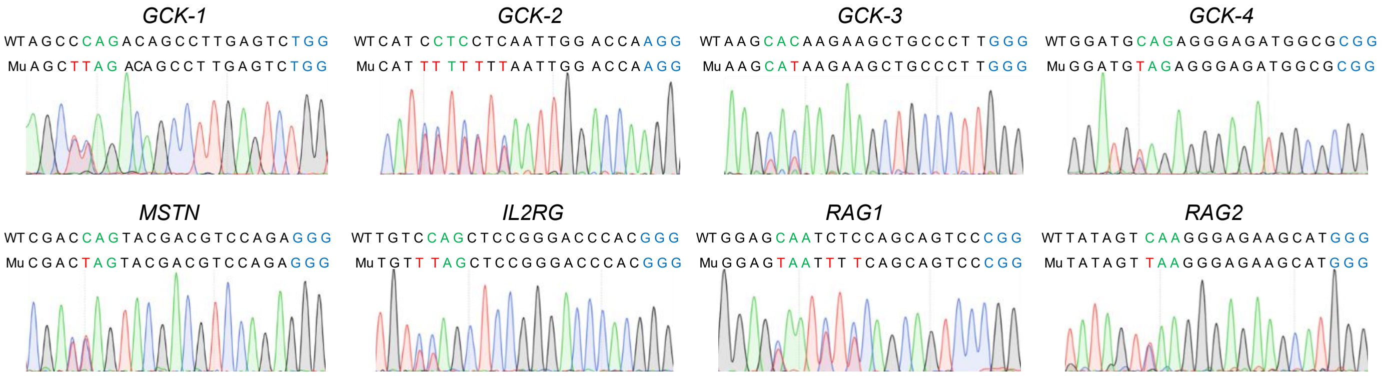

a

Target genes	Single gene mutant (%)	Two genes mutant (%)	Three genes mutant (%)
RAG1/RAG2	*RAG1*: 7/16 (43.8%)<br>*RAG2*: 9/16 (56.3%)	*RAG1/RAG2*: 6/16 (37.5%)	-
RAG1/RAG2/IL2RG	*RAG1*: 10/20 (50.0%)<br>*RAG2*: 9/20 (45.0%)<br>*IL2RG*: 5/20 (25.0%)	*RAG1/RAG2*: 7/20 (35.0%)<br>*RAG1/IL2RG*: 3/20 (15.0%)<br>*RAG2/IL2RG*: 5/20 (25.0)	*RAG1/RAG2/IL2RG*: 3/20 (15%)
GCK-2/GCK-3	*GCK-2*: 17/22 (77.3%)<br>*GCK-3*: 10/22 (45.5%)	*GCK-2/GCK-3*: 10/22 (45.5%)	-

b

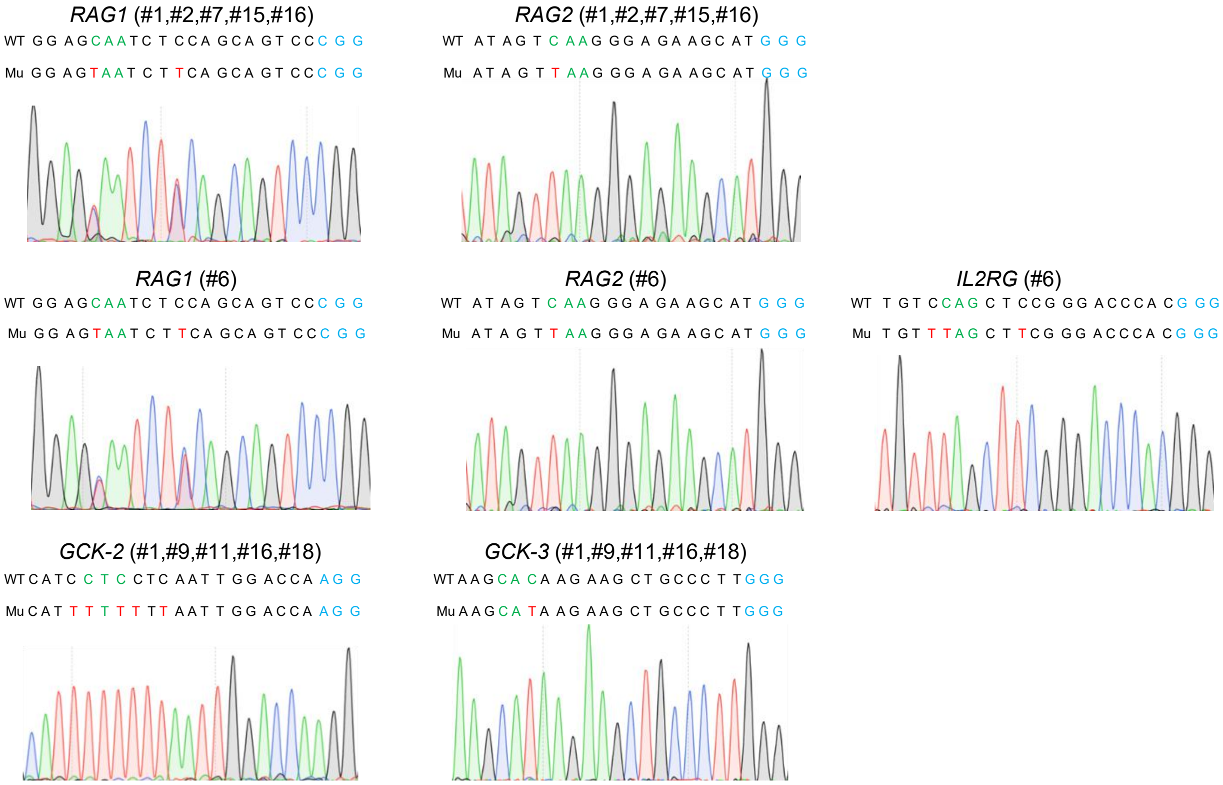

c

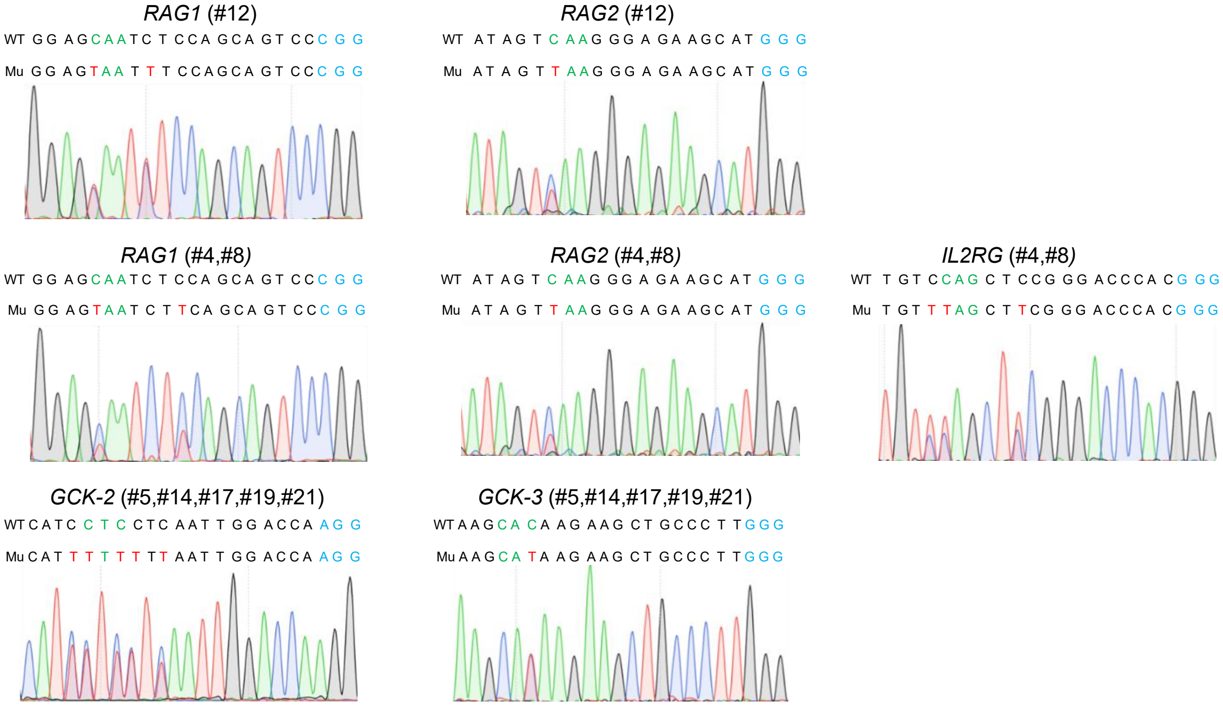

a

No. of zygotes injected	No. of zygotes transferred	Birth	Mutation	Biallelic mutation
56	56	17	4	3

b

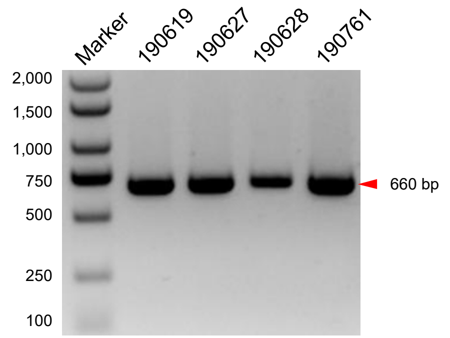

c

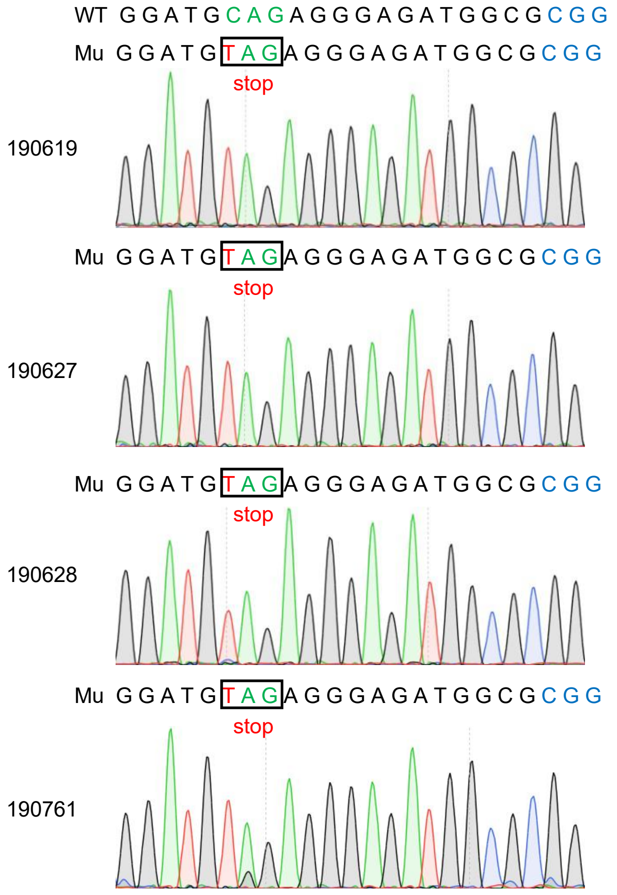

d

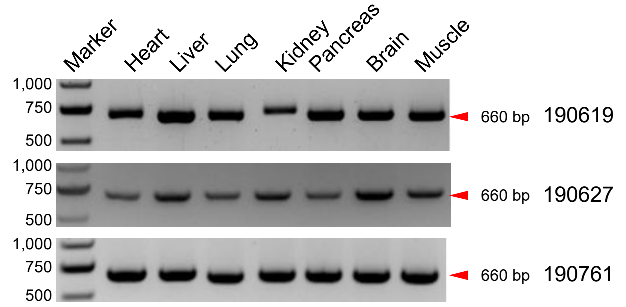

e

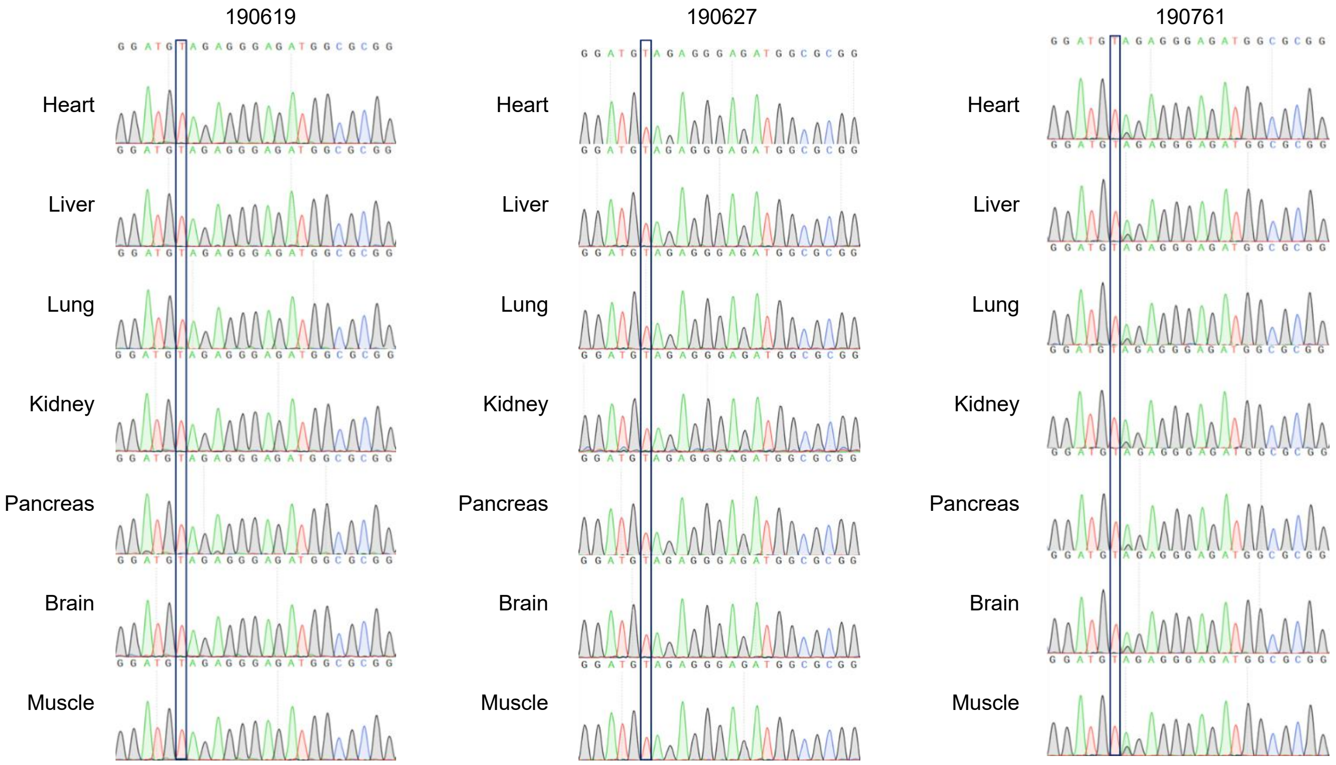

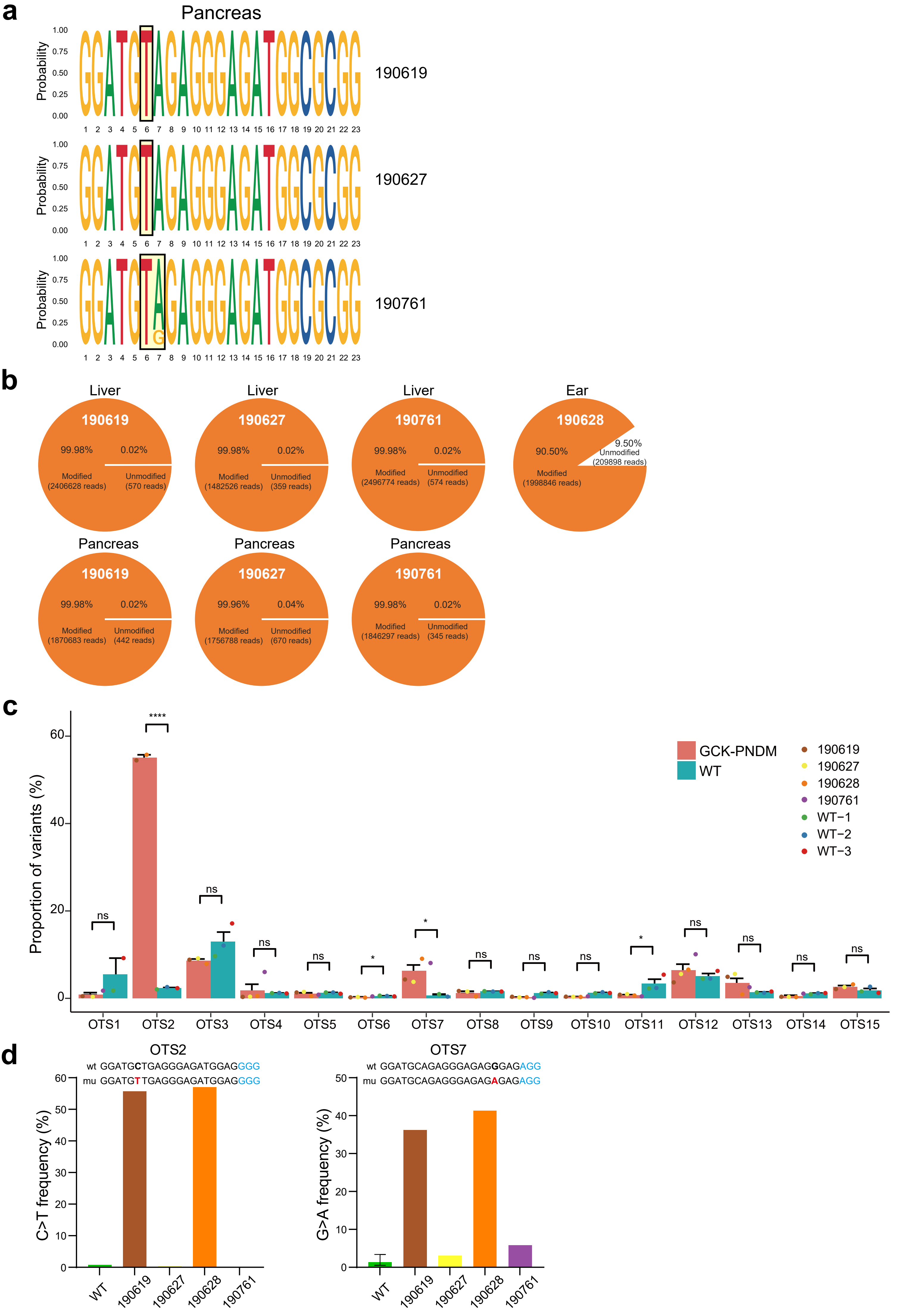

a
Pancreas
Probability
1.00
0.75
0.50
0.25
0.00
1 2 3 4 5 6 7 8 9 10 11 12 13 14 15 16 17 18 19 20 21 22 23
190619
190627
190761
b
Liver
190619
99.98% Modified (2406628 reads)
0.02% Unmodified (570 reads)
Liver
190627
99.98% Modified (1482526 reads)
0.02% Unmodified (359 reads)
Liver
190761
99.98% Modified (2496774 reads)
0.02% Unmodified (574 reads)
Ear
190628
90.50% Modified (1998846 reads)
9.50% Unmodified (209898 reads)
Pancreas
190619
99.98% Modified (1870683 reads)
0.02% Unmodified (442 reads)
Pancreas
190627
99.96% Modified (1756788 reads)
0.04% Unmodified (670 reads)
Pancreas
190761
99.98% Modified (1846297 reads)
0.02% Unmodified (345 reads)
c
Proportion of variants (%)
0
20
40
60
GCK-PNDM
WT
190619
190627
190628
190761
WT−1
WT−2
WT−3
OTS1 ns
OTS2 ****
OTS3 ns
OTS4 ns
OTS5 ns
OTS6 *
OTS7 *
OTS8 ns
OTS9 ns
OTS10 ns
OTS11 *
OTS12 ns
OTS13 ns
OTS14 ns
OTS15 ns
d
OTS2
wt GGATGCTGAGGGAGATGGAGGGG
mu GGATGTTGAGGGAGATGGAGGGG
C>T frequency (%)
0
10
20
30
40
50
60
WT
190619
190627
190628
190761
OTS7
wt GGATGCAGAGGGAGAGGGAGAGG
mu GGATGCAGAGGGAGAGAGAGAGG
G>A frequency (%)
0
10
20
30
40
50
WT
190619
190627
190628
190761

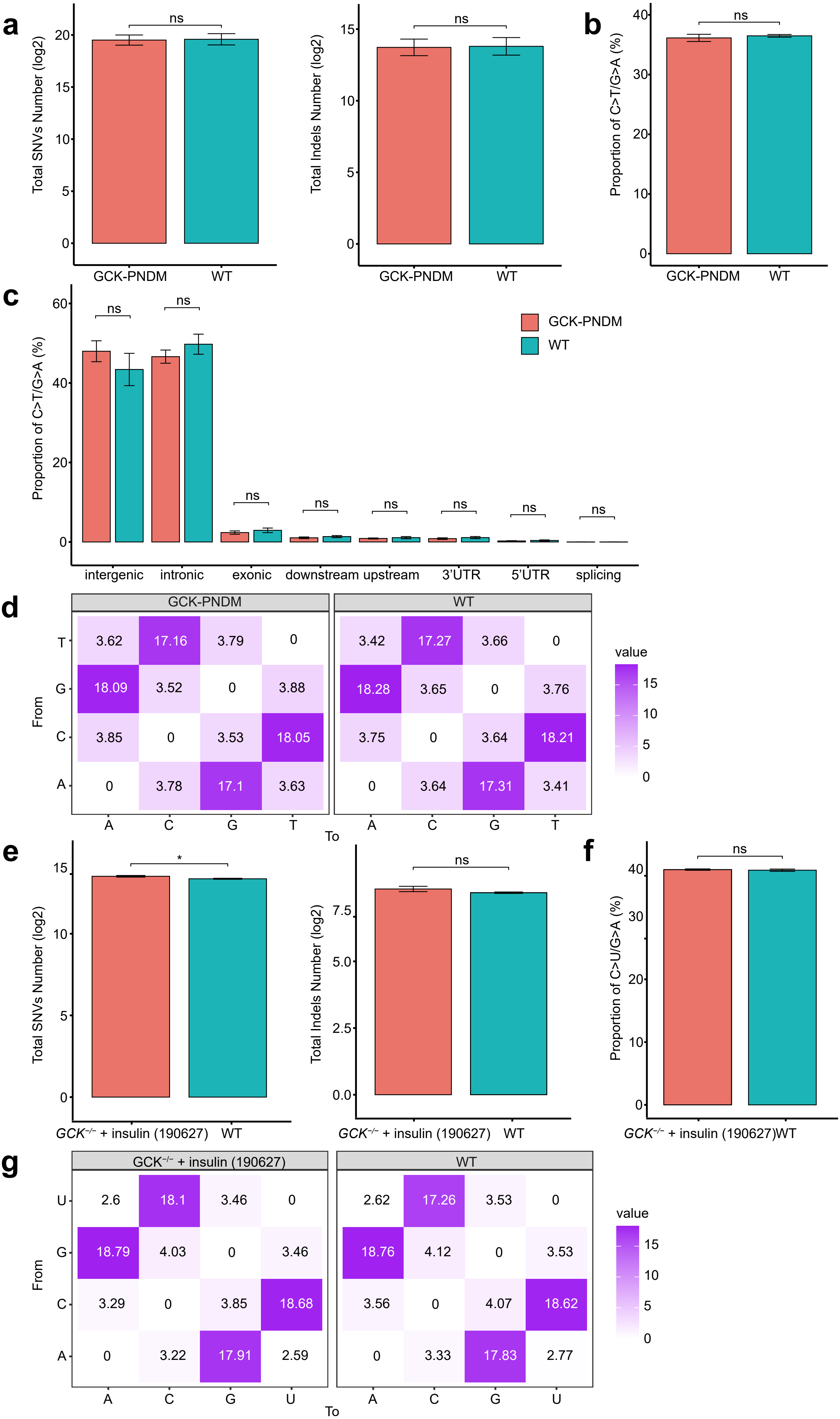
a
ns
Total SNVs Number (log2)
GCK-PNDM
WT
Total Indels Number (log2)
b
Proportion of C>T/G>A (%)
c
intergenic
intronic
exonic
downstream
upstream
3'UTR
5'UTR
splicing
d
From
To
value
3.62
17.16
3.79
0
18.09
3.52
0
3.88
3.85
0
3.53
18.05
0
3.78
17.1
3.63
3.42
17.27
3.66
0
18.28
3.65
0
3.76
3.75
0
3.64
18.21
0
3.64
17.31
3.41
e
*
GCK−/− + insulin (190627)
f
Proportion of C>U/G>A (%)
g
2.6
18.1
3.46
0
18.79
4.03
0
3.46
3.29
0
3.85
18.68
0
3.22
17.91
2.59
2.62
17.26
3.53
0
18.76
4.12
0
3.53
3.56
0
4.07
18.62
0
3.33
17.83
2.77

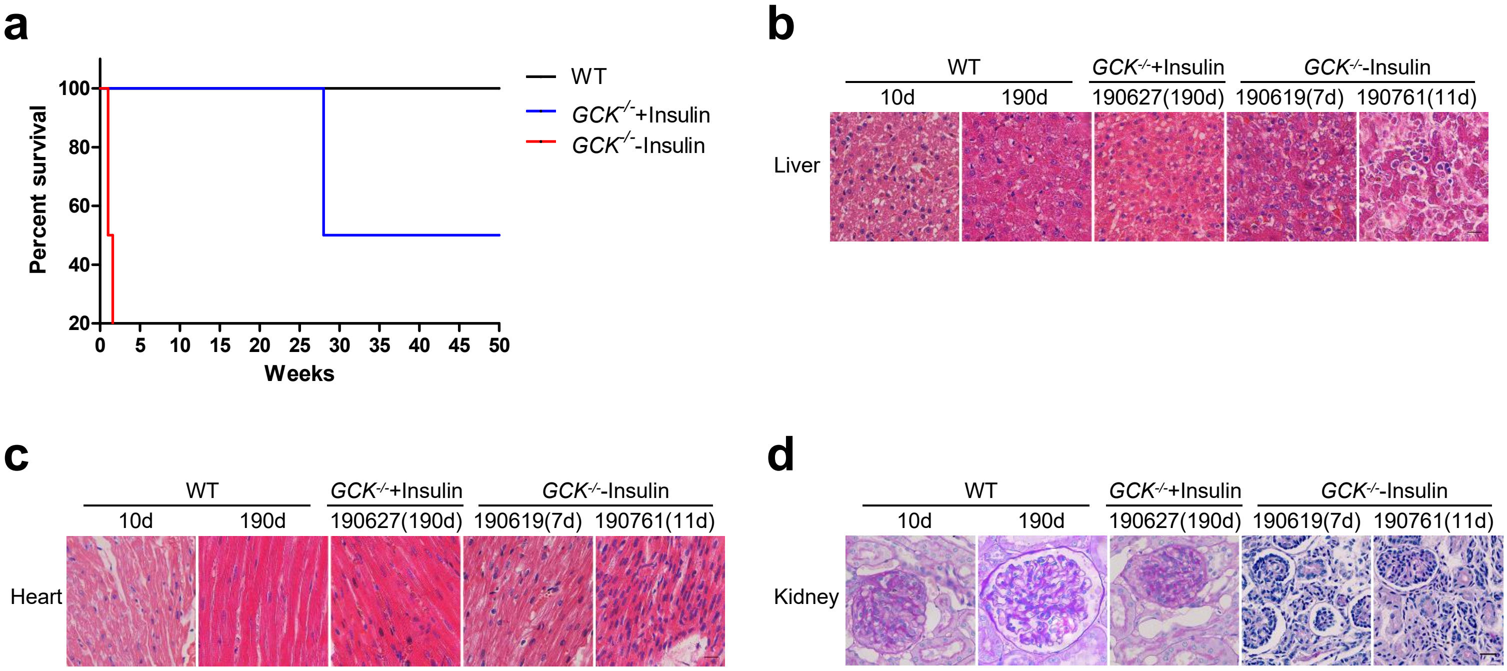
a
Percent survival
100
80
60
40
20
0 5 10 15 20 25 30 35 40 45 50
Weeks
WT
GCK-/-+Insulin
GCK-/--Insulin
b
WT
GCK-/-+Insulin
GCK-/--Insulin
10d
190d
190627(190d)
190619(7d)
190761(11d)
Liver
c
WT
GCK-/-+Insulin
GCK-/--Insulin
10d
190d
190627(190d)
190619(7d)
190761(11d)
Heart
d
WT
GCK-/-+Insulin
GCK-/--Insulin
10d
190d
190627(190d)
190619(7d)
190761(11d)
Kidney

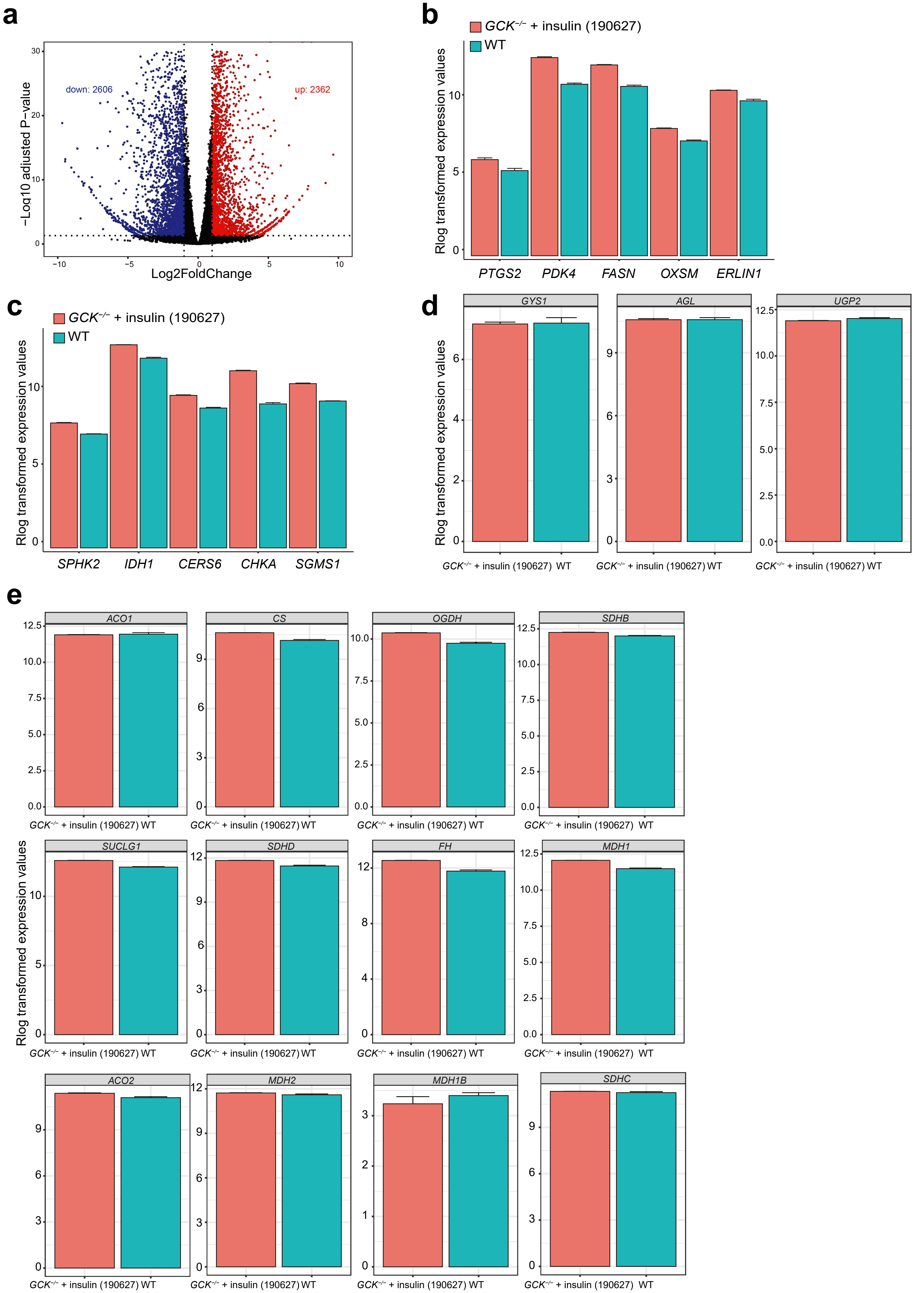

a
down: 2606
up: 2362
−Log10 adjusted P−value
Log2FoldChange
b
GCK−/− + insulin (190627)
WT
Rlog transformed expression values
PTGS2
PDK4
FASN
OXSM
ERLIN1
c
GCK−/− + insulin (190627)
WT
Rlog transformed expression values
SPHK2
IDH1
CERS6
CHKA
SGMS1
d
GYS1
AGL
UGP2
Rlog transformed expression values
GCK−/− + insulin (190627) WT
e
ACO1
CS
OGDH
SDHB
SUCLG1
SDHD
FH
MDH1
ACO2
MDH2
MDH1B
SDHC
Rlog transformed expression values
GCK−/− + insulin (190627) WT

Fig. S1 Effectiveness of C-to-T conversation at multiple genes/loci in canine embryonic fibroblasts (CEFs) by using BE3 system.

a Target-site sequences and efficiencies of base editing within the *GCK* (4 sites), *MSTN*, *IL2RG*, *RAG1*, and *RAG2*. Target sequence (black), protospacer adjacent motif (PAM) region (blue), target sites (green).

b Representative sequencing chromatograms with homozygous mutations at target-sites of *GCK* (*GCK-2* and *GCK-4*), *MSTN*, *IL2RG*, *RAG1*, and *RAG2* in CEFs. Target sequences (black), protospacer adjacent motif (PAM) region (blue), target sites (green), and mutant sites (red). WT wild-type, Mu mutant.

c Representative sequencing chromatograms with heterozygous mutations at target-sites of *GCK* (*GCK-1*, *GCK-2*, *GCK-3*, and *GCK-4*), *MSTN*, *IL2RG*, *RAG1*, and *RAG2* in CEFs. Target sequences (black), protospacer adjacent motif (PAM) region (blue), target sites (green), and mutant sites (red). WT wild-type, Mu mutant.

Fig. S2 Base editing at multiple genes/loci simultaneously in CEFs by using BE3 system.

a Summary of multiple genes or loci base editing efficiencies by BE3 in CEFs.

b Representative sequencing chromatograms of clones (#1, #2, #7, #15, and #16) edited with heterozygous mutation at *RAG1* and homozygous mutation at *RAG2* simultaneously in CEFs; Clone (#6) edited with heterozygous mutation at *RAG1*, homozygous mutations at *RAG2* and *IL2RG* simultaneously in CEFs; clones (#1, #9, #11, #16, and #18) edited with homozygous mutations at *GCK-2* and *GCK-3* simultaneously in CEFs. Target sequences (black), protospacer adjacent motif (PAM) region (blue), target sites (green), and mutant sites (red). WT wild-type, Mu mutant.

c Representative sequencing chromatograms of clones with heterozygous mutations at multiple sites/loci simultaneously in CEFs. Target sequences (black), protospacer adjacent motif (PAM) region (blue), target sites (green), and mutant sites (red). WT wild-type, Mu mutant. Numbers in brackets represent positive clones in multiple-gene combination test.

Fig. S3 Generation of GCK-PNDM dogs with BE3 system.

a Summary of generation of GCK-PNDM dogs by delivering BE3 system *via* zygote injection.

b-c Genotypes identification by PCR reaction (**b**) and Sanger sequencing (**c**) using genomic DNA isolated from ear punch tissue of the GCK-PNDM dogs. M DL2000 DNA marker.

d-e Genotypes identification by PCR reaction (**d**) and Sanger sequencing (**e**) using genomic DNA isolated from heart, liver, lung, kidney, pancreas, brain, and muscle tissues of the GCK-PNDM dogs. M DL2000 DNA marker.

Fig. S4 Chimerism and sgRNA-dependent DNA off-target analysis of the GCK-PNDM dogs.

a Sequence motif of pancreas from the GCK-PNDM dogs (n=3, 190619, 190627, and

190761).

b Deep sequencing analysis of modified ratio of different tissues and organs from GCK-PNDM dogs.

c Comparison of the mutation frequency of 15 potential off-target sites (OTSs) in *GCK* gene between the WT and GCK-PNDM dogs. WT (n=3), GCK-PNDM (n=4, 190619, 190627, 190628, and 190761). $^{*}P < 0.1$; $^{****}P < 0.0001$; ns, not significant.

d Proportion of C>T/G>A mutations of the 2 off-target sites in the WT and GCK-PNDM groups. WT (n=3), GCK-PNDM (n=4, 190619, 190627, 190628, and 190761). Target sequences (black), protospacer adjacent motif (PAM) region (blue), target sites (green), and mutant sites (red). WT wild-type, Mu mutant.

Fig. S5 sgRNA-independent DNA off-target and RNA off-target analysis of the GCK-PNDM dogs.

a-b Whole genome sequencing (WGS) analysis showed that no significant difference in the number of *de novo* SNVs, indels (**a**), and proportion of C>T/G>A (**b**) in the WT and GCK-PNDM groups. WT (n=3), GCK-PNDM (n=3, 190619, 190627, and 190628). ns, not significant.

c Distribution of C>T/G>A mutations in the different regions in the WT and GCK-PNDM groups. WT (n=3), GCK-PNDM (n=3, 190619, 190627, and 190628). ns, not significant.

d Distribution of DNA mutation types in the WT and GCK-PNDM groups. WT (n=3), GCK-PNDM (n=3, 190619, 190627, and 190628). The number in each cell indicated the proportion of a certain type of mutation among all mutations.

e-f RNA-seq analysis showed that no difference in the number of *de novo* SNVs, indels (**e**), and proportion of C>U/G>A (**f**) in liver samples from the WT and GCK-PNDM groups. WT (n=1), GCK-PNDM (n=1, 190627). $^{*}P < 0.1$; ns, not significant.

g Distribution of DNA mutation types in liver samples from the WT and GCK-PNDM groups. WT (n=1), GCK-PNDM (n=1, 190627). The number in each cell indicated the proportion of a certain type of mutation among all mutations.

Fig. S6 Characteristics of the GCK-PNDM dogs.

a Survival curve of the WT and GCK-PNDM dogs.

b-d Histological analysis of WT (10d and 190d), $GCK^{-/-}$ + insulin (190d), and $GCK^{-/-}$ - insulin (7d and 11d) dogs. H&E staining of liver (**b**) and heart sections (**c**) showed no observable histological abnormalities. PAS staining of kidney sections (**d**) for analysis of glycogen (purple color, white arrow) synthesis. Scale bar: 80 μm

Fig S7 Differential expression analysis of the insulin treated GCK-PNDM dog (190627).

a The volcano plot of differential expressed genes. Up-regulated genes (red spots), down-regulated genes (blue spots). $P < 0.05$.

b-c Bar plots of representative up-regulated genes related to lipid transport fatty acid biosynthetic process (**b**) and lipid biosynthetic process (**c**) between the insulin treated GCK-PNDM and WT dogs.

d-e Bar plots of representative genes involved in glycogen metabolic process (**d**) and tricarboxylic acid (TCA) cycle processes (**e**) between WT and the insulin treated GCK-PNDM dogs. $P < 0.05$ was considered statistically significant.

Methods

Animals

Female beagle dogs aged from about 1 to 5 years old were used in this study for embryo donors or surrogate mothers. Dogs in estrus stage were purchased from the lab animal center. All experiments involving animals were approved by the Guidelines for the Care and Use Committee of Beijing SINOGENE Biotechnology Co., Ltd.

Vector construction and RNA transcription *in vitro*

The synthesized BE3 fragments were cloned into the pCS2 vector with the T7 promoter and into the pcDNA3.1 (+) vector under the CMV promoter. Construction of pU6-sgRNA or pT7-sgRNA cloning vector was performed as described previously[1]. Briefly, pU6-sgRNAs were designed by N20-NGG rule and pT7- *GCK*-sgRNA was designed by G-N19-NGG rule with the sequences as follows: ataGGATGCAGAGGGAGATGGCGgt and taaaacCGCCATCTCCCTCTGCATC. The synthesized sgRNAs were annealed to double-stranded DNAs and then cloned into the BbsI-digested pU6-sgRNA or pT7-sgRNA cloning vector. For transcription of RNA *in vitro*, both BE3 and sgRNA vectors under the T7 promoter were synthesized using RNA Synthesis Kit (NEB) and then purified by RNeasy MinElute Cleanup Kit (74204, Qiagen). The purified mRNA of BE3 and sgRNA were diluted to 400 and 40 ng/μL with RNase-free water, respectively. The mRNA of BE3 and sgRNA were stored at −80 °C and mixed in a 1:1 ratio before microinjection at a final concentration of 200 and 20 ng/μL, respectively.

Estrus identification by progesterone levels

Estrus identification were conducted as described previously[2]. Briefly, female dogs in estrus stage were observed with the color change of vaginal bleeding daily. Vaginal smears were performed with Giemsa staining and detected under a light microscope for preliminary identification. Blood samples (2 mL) were collected daily near the

ovulation date by cephalic venipuncture and serum was retrieved by centrifuging at 300 × g for 15 min. The day when the serum progesterone level initially increased to 4.0-7.5 ng/mL was considered as the day for ovulation.

Zygotes collection and injection

Zygotes collection and injection were performed as previous described [2]. Briefly, laparotomy was conducted the day after natural mating within 18 h to 24 h. The oviduct was exposed by a medioventral line laparotomy for embryo flushing. About 10 mL of the tissue culture medium-199 supplemented with 10% FSC were passed through the oviductal lumen and collected into a sterile plastic tube. The collected presumptive zygotes were transported to the laboratory and injected with a mixture of BE3 mRNA (200 ng/μL) and *GCK* sgRNA (20 ng/μL) after removing cumulus cells with 0.1% (w/v) hyaluronidase.

Embryos Transfer and Pregnancy Diagnosis

Embryos injected with RNA mixture were transferred into the oviduct of recipient females through allo-transplantation or auto-transplantation. Briefly, the recipient females were aseptically prepared for surgery and the reproductive tracts were exposed through a caudal ventral incision. Embryos were transferred into the ampullar by feeding a 3.5F Tom cat catheter (Kendall) through the oviduct. Pregnancy diagnosis were performed by using ultrasound scanner with an attached 7.0 MHZ linear probe after 30 days of transplantation. The pregnant dogs were monitored with more care.

Canine embryonic fibroblast cell culture and transfection

Canine embryonic fibroblast cells (CEFs) were cultured and transfected as previous described [2]. Briefly, pCMV-CBE3 and pU6-sgRNA plasmids were co-transfected into CEFs using Neon® Transfection System (Invitrogen). About 1×10^6 cells were electroporated with 6 μg pCMV-CBE3 and 3 μg pU6-gRNA plasmids under 1350 V, 30 ms, 1 pulse transfection condition. The electroplated cells were recovered for 24 h

and then divided into 10 cm diameter culture dishes for selection. The next day, 300 μg/mL of G418 (Merck) was added into the culture media for cell clone screening. After 10 days screening, the individual cell clones were picked up and about 20% of each clone cells were lysed in 10 μL lysis buffer (0.45% NP-40 plus 0.6% Proteinase K) at 56 °C for 60 min. The lysate was then used as a template for PCR.

DNA extraction and sequencing

The genomic DNA was extracted from the ear punch tissues of all the 4 PNDM dogs (190619, 190627, 190628, and 190761) and 3 wild type (WT) dogs by Cell Genome Extraction kit (DP304-03, Tiangen Biotech) according to the manufacturer's instructions. Sanger sequencing after PCR was performed with primers as follows: F: 5'-GTGCCTCCCTCCTCCCCAT-3'; R: 5'-CACAAAGCCAGTTTCCCCA-3'.

Measurement of body weights, blood glucose and injection of insulin

Body weights and blood glucose concentrations of WT and GCK-PNDM dogs were measured daily at early age or weekly at old age. Blood glucose levels of the dogs were detected through ear vein by using blood glucose test paper and reading out the results from blood glucose meter (GA-3, Sinocare) before eating or insulin injection. Two of the GCK-PNDM dogs (190627 and 190628) were injected with insulin twice a day. And the dosage of insulin injected was gradually increased when the body weight increased.

Histological analysis

Tissues of kidney, liver and heart from WT and GCK-PNDM dogs were fixed with 4% paraformaldehyde, then embedded in paraffin and sectioned. Sections of kidney, liver and heart were stained with hematoxylin and eosin and examined under the microscopy. For detection of glycogen formation, sections of kidney and liver were stained by periodic acid-Schiff (PAS) staining and checked under the microscopy. Sections of heart from WT and GCK-PNDM dogs were stained by Masson's trichrome staining to check fibrosis in heart. For examination of lipid in liver,

cryosections were stained with the Oil Red *O* following counterstained with hematoxylin.

Whole-genome sequencing and bioinformatics analysis of single nucleotide variants

The genomic DNA from the 3 GCK-PNDM dogs (190619, 190627, and 190628) and 3 WT dogs were used in the WGS analysis. The 3 WT dogs were used as a control in the analysis of the genome-wide *de novo* mutations. WGS was performed at mean coverages of 30× by Illumina Novaseq. Quality control was performed by removing adapter sequences and reads with low complexity or of low quality. Bwa (version 0.7.15-r1140) was used for mapping sequenced reads to canine genome from NCBI (GCF_014441545.1). Samtools (version 1.3.1) was used for processing aligned bam files. Duplicated reads in the bam file were removed by Picard (version 2.25.0).

During the downstream analysis, in order to ensure the reliability of the WGS sequencing results, three softwares were used for detecting *de novo* SNVs, namely gatk(version 4.2.0.0), freebayes(version 1.3.4) and bcftools(version 1.4). Only SNVs detected by at least two softwares were considered as true mutations. The common SNVs that simultaneously appeared in WT and PNDM dogs were considered as background noise and thus were eliminated in further analysis. Vcftools (version 0.1.13) and snpEff (version 5.0e) was used for processing vcf files, and custom R scripts was used for drawing the figures.

Deep Sequencing and bulk RNA-seq analysis

Genomic DNA from livers and pancreases of WT and GCK-PNDM dogs (190619, 190627, and 190761) or from ear skin of GCK-PNDM dog (190628) were extracted. The target sites were amplified for 30 cycles with 98 ℃ 10 s, 58 ℃ 30 s, 72 ℃ 8 s by Q5 DNA polymerases (Q5 High-Fidelity 2× Master Mix, M0492S, NEB). The PCR products were then used as a template for another amplification with different index primers of different samples at 98 ℃ 10 s, 58 ℃ 30 s, 72 ℃ 8 s for 12 cycles. PCR

products of all the samples were gel-purified using the Gel Extraction Kit (HiPure Gel Pure DNA Mini Kit, D1001-03, Magen). Following concentration determination, all the samples were mixed together to make a DNA library and then used for deep sequencing by Illumina Hiseq2000. For bulk RNA-seq analysis, liver samples of WT and GCK-PNDM (190627) dogs were collected and used for RNA extraction (RaPure Total RNA Micro Kit, R4012-03, Magen). The RNA-seq libraries were prepared by Zhejiang ANNOROAD Biotechnology Ltd and then sequenced on Illumina HiSeq1500 or 2500 according to the manufacturer's instructions.

Analysis of sgRNA-dependent off-target effects

Potential off-target sites (OTSs) of the *GCK* sgRNA were predicted by "Genome Target Scan" (GT-Scan) online tool. Potential OTSs with 2 or 3 nucleotide mismatches at the target site were chosen for PCR amplification by using genomic DNA extracted from the ear punch tissues of 4 PNDM dogs (190619, 190627, 190628, and 190761) and 3 WT dogs as templates. The target sites were amplified for 30 cycles with 95 °C 15 s, 55 °C 15 s, 72 °C 4 s by DNA polymerases (2×Rapid Taq Master Mix, P222-03, Vazyme). The PCR products were then used as a template for another amplification with different index primers of different samples at 95 °C 15 s, 55 °C 15 s, 72 °C 4 s for 12 cycles. All the PCR products were no more than 200 bp and used for amplicon library construction. The sequences of all the tested OTSs and primers for amplification are listed in Supplementary Table 1. During the downstream analysis, the variants that simultaneously appeared in 3 WT dogs were considered as background noise and thus eliminated in further analysis.

Statistical analysis

All data were performed at least three biological replicates. The differences of groups were analyzed with Wald test. A probability of $p < 0.05$ was considered statistically significant. *$P < 0.1$, **$P < 0.01$, ***$P < 0.001$, ****$P < 0.0001$, ns, not significant.

1 Feng, C. *et al.* Generation of ApoE deficient dogs via combination of embryo

injection of CRISPR/Cas9 with somatic cell nuclear transfer. *J Genet Genomics* **45**, 47-50, doi:10.1016/j.jgg.2017.11.003 (2018).
2 Zou, Q. *et al.* Generation of gene-target dogs using CRISPR/Cas9 system. *J Mol Cell Biol* **7**, 580-583, doi:10.1093/jmcb/mjv061 (2015).

Supplementary Table 1

Table S1. Sequences of the tested potential off-target sites and primers for amplification.

OTSs	Sequences	NO. of mismatch (position)	Primer sequences (5'-3')	PCR product length	Sequences compared with WT			
					190619	190627	190628	190761
OTS1	GGGTGCAGA GGGAGATGG AGAGG	2 (35)	AGTGGGGGAAG CCCATCT TCAGGGTCCTGGA ATCAAGC	170	wt[1]	wt	wt	wt
OTS2	GGATGCTGA GGGAGATGG AGGGG	2 (8)	GGCCAAGGACCTT GTTATTTGTG GAGCATTTGCTGC ACATGCC	153	mu[2]	wt	mu	wt
OTS3	GGATGTAGA GGGAGATGG GGGGG	2 (2)	GCTGCCAGTACAC AGGCGGGAA TGAGCCCTCCATG GCCTCCT	152	wt	wt	wt	wt
OTS4	GGATGCAGA GGGAGAGG GAGAGG	2 (21)	ATGTCTGGACTAC CCACCCT GATCAAGCCCCAT GTCAGGC	161	wt	wt	wt	wt
OTS5	GGATGCAGA GGGAGAGG GAGAGG	2 (27)	CTTTCTACCCTACA GAAGTGGCT GATTCCAGCGTCC TGGGACT	167	wt	wt	wt	wt
OTS6	GGATGCAGA GGGTGAGGG CGAGG	2 (30)	GTTGTGGCAGCTG CACGTAT GGAAATTCCAGCT GGGTGTATGC	159	wt	wt	wt	wt
OTS7	GGATGCAGA GGGAGAGG GAGAGG	2 (1)	AGATTGATTCATTT TGAGTTACA CTCAGGGTTGTGA GACTGAG	161	mu	wt	mu	mu
OTS8	GGATGCAGA GGGAGACG GAGGGG	2 (10)	GTGTGGGTAGAGA TCGGACGAG GAACCTCCTGAGG GTCTCACTT	164	wt	wt	wt	wt
OTS9	TGATGGAGA TGGAGATGG CGAGG	3 (30)	GAACTGCTTGAAG ATCGGGTGG GAATGGCAGTGTG	166	wt	wt	wt	wt

			CAGTGCA					
OTS10	GGGGGCAG AGGGAGATG GAGAGG	3 (2)	GTGTACTACTTTGA AACACGTGC GCTCAGGTCCTGA GAGGCAG	173	wt	wt	wt	wt
OTS11	GGATGCAGA GAGAGAAG GGGAGG	3 (12)	GGGTCACACCTTA GCTTGAGACT GGCTGTCTTGCTTC TGCCCA	165	wt	wt	wt	wt
OTS12	GGATGCAGA GAGAGAGG GGGAGG	3 (12)	ACCTACAAAGCAC CAACCAAG GATCCTAGAGTCC TGGGATCCA	185	wt	wt	wt	wt
OTS13	GGGGGCAG AGGGAGATG GAGAGG	3 (14)	GAGGCATTGGTAT CATTAAGCA GATGTCAGTATTGT GAGATCG	167	wt	wt	wt	wt
OTS14	GGATGCAGA GGAAGGTGG GGAGG	3 (17)	GGGCAGAAGAGTT GTAAGAAGACG TCATTGCTCAGGT GCCTGG	160	wt	wt	wt	wt
OTS15	GGATGCAGA GGGGGAAG GAGAGG	3 (36)	GAAGGAAAGGAG TGGAAG TGTGTGGGGCTCT GTGCT	187	wt	wt	wt	wt

[1]wt: wild type sequence

[2]mu: mutant sequence

Mismatched nucleotides (orange), protospacer adjacent motif (PAM) region (blue).
